# Characteristics of Older Patients with Heart Failure Readmitted due to Acute Exacerbations within the Past Year

**DOI:** 10.1298/ptr.E10187

**Published:** 2022-12-28

**Authors:** Yuki NONAKA, Takayuki OIKE, Shinichiro TANAKA, Kazuyuki TABIRA

**Affiliations:** ^1^Graduate School of Health Science, Kio University, Japan; ^2^Musashigaoka Clinical Research Center, Musashigaoka Hospital, Medical Corporation Tanakakai, Japan; ^3^Department of Rehabilitation, Musashigaoka Hospital, Medical Corporation Tanakakai, Japan; ^4^Department of Rehabilitation, Kyushu University of Nursing and Social Welfare, Japan

**Keywords:** Heart failure, Older adults, Readmission, Short Physical Performance Battery, Brain natriuretic peptide

## Abstract

Objective: We aimed to examine the relationship between physical performance and readmission among older patients with heart failure (HF) over the past year. Methods: This retrospective cohort study included 325 patients with HF who were aged ≥65 years and were hospitalized for acute exacerbation between November 2017 and December 2021. We investigated age, sex, body mass index, length of hospital stay, initiation of rehabilitation, New York Heart Association (NYHA) class, Charlson comorbidity index (CCI) score, medications, cardiac/renal function, nutrition, maximal quadriceps isometric strength, grip strength, and Short Physical Performance Battery (SPPB) score. Data were analyzed using the χ^2^ test, Mann–Whitney U test, and logistic regression analysis. Results: Altogether, 108 patients met the inclusion criteria and were divided into the non-readmission (n = 76) and readmission (n = 32) groups. The readmission group exhibited longer hospital stay, more severe NYHA class, higher CCI score, higher brain natriuretic peptide (BNP) levels, lower muscle strength, and lower SPPB score compared to the non-readmission group. In the logistic regression model, BNP level and SPPB score were independent factors associated with readmission. Conclusion: BNP level and SPPB score were associated with readmission in patients with HF within the past year.

**T**he number of older adults is increasing throughout the world. In Japan, it has been reported that older adults account for 28.4% of the total population^1,2)^. An increase in the incidence of heart failure (HF) due to aging has become a major problem. According to a previous study, the estimated prevalence of HF in the United States is 5.1 million, which is predicted to increase to 8 million by 2030^3)^. A previous Japanese study estimated that the total number of patients with left ventricular dysfunction in Japan (both systolic and diastolic dysfunction) would increase from 979,000 in 2005 to 1,284,000 by 2030^4)^. Patients with HF encounter problems such as increased readmission rates, increased medical costs, and multiple short-term hospitalizations^5,6)^.

HF is a major cause of hospitalization and death among older adults worldwide^7)^. Factors related to readmission of patients with HF include exacerbation of HF as well as various conditions such as respiratory diseases, kidney diseases, psychiatric diseases, bone and joint diseases, and diabetes^8,9)^. Shiraishi et al. reported that 5.4% of the patients with HF were readmitted within 30 days, while 26.2% of them were readmitted within 1 year^10)^. In addition, according to the Rationale and Design of Japanese Cardiac Registry of Heart Failure in Cardiology (JCARE-CARD), the readmission rate among patients with HF was as high as 35% within 1 year^11)^, indicating a high risk of readmission among these patients within 1 year. Furthermore, patients with HF have to bear high medical costs, and it has been reported that patients with higher serum N-terminal pro-brain natriuretic peptide levels have to bear higher medical costs associated with hospitalization^12,13)^. Thus, reducing the readmission rate of patients with HF would reduce the medical costs.

In recent years, many reports have shown that the Short Physical Performance Battery (SPPB) is useful for functional evaluation of older patients, and a meta-analysis revealed that an SPPB score of <10 points is an excellent predictor of all-cause mortality^14–16)^. In addition, the SPPB score has also been reported to predict future decline in the activities of daily living (ADLs) among older adults^17)^. The SPPB score at discharge may be useful in predicting future decline in the ADLs and readmission among patients with HF.

Therefore, we hypothesized that the SPPB score may be a strong predictor of readmission among patients with HF, and patients with HF having low SPPB scores would have been repeatedly hospitalized and discharged within the past year.

## Methods

### Participants

The present study was a single-center cross-sectional study. This retrospective cohort study included 325 patients with HF admitted to a community-based hospital in Kumamoto, Japan, for acute exacerbations from November 2017 to December 2021. An experienced cardiologist diagnosed acute decompensated HF based on the Framingham criteria and Japanese guidelines^18)^. The inclusion criteria were age ≥65 years and ability to walk with assistance at discharge. Patients who did not consent to participation, patients who died during hospitalization, patients who were transferred to another hospital, and patients in a poor general condition that could have caused difficulties while conducting the measurements were excluded from this study. All participants in this study underwent cardiac rehabilitation.

### Investigations

Patient characteristics and clinical parameters at discharge such as age, sex, body mass index (BMI), length of hospital stay, initiation of rehabilitation, New York Heart Association (NYHA) class, Charlson comorbidity index (CCI) score, comorbidities, medications, left ventricular ejection fraction (LVEF), brain natriuretic peptide (BNP), creatinine (Cr), blood urea nitrogen (BUN), estimated glomerular filtration rate (eGFR), hemoglobin (Hb), albumin (Alb), geriatric nutritional risk index (GNRI) score, maximal quadriceps isometric strength (QIS), grip strength, and SPPB score were investigated by reviewing the medical records. LVEF was not measured at discharge in many patients. Therefore, we used the data at admission. According to the Japanese guidelines, it has been shown that 60%–80% of the patients with HF having reduced ejection fraction and 60%–90% of the patients with HF having preserved ejection fraction have HF with unchanged LVEF^19)^.

### Assessment of the NYHA class, maximal QIS, and grip strength

We evaluated the maximal QIS, grip strength, and NYHA class at discharge. The NYHA class was determined by an experienced cardiologist and a physiotherapist specializing in cardiac rehabilitation.

The maximal QIS was measured using a handheld dynamometer (Mobie MT-100; SAKAI, Tokyo, Japan). Patients sat on a chair with their arms folded and knees flexed at 90°. The pad position was established at 30 mm above the lateral malleolus in all patients. Knee extension strength was measured twice on both the right and the left sides with maximum force. We used the maximum value multiplied by the leg length and divided by the body weight (Nm/kg).

Grip strength was measured to evaluate the upper limb muscle strength. A digital grip force meter (Grip D T.K.K. 5401; Takei Scientific Instruments, Niigata, Japan) was used to measure grip strength. The measurements were performed according to the method recommended by the Ministry of Education, Culture, Sports, Science, and Technology^20)^. However, since many patients were unstable in the standing posture, measurements were performed in the sitting position. The maximum grip strength was measured twice on each side and the highest value was used.

### Assessment of the SPPB score

The SPPB score was assessed based on standing balance, 4-m walking velocity, and time to rise five times from the seated position^14)^. Participants were asked to hold each of the three standing positions, namely closed-leg standing, semi-tandem stance (heel of one foot touching the base of the toe of the other foot), and full tandem stance (heel of one foot directly touching the other toe) for 10 s during the assessment for balance. Walking velocity was measured using a 4-m walk performed at the patient’s usual pace. Participants were allowed to use canes or walkers. For measurement of time to rise from the seated position, participants sat on a chair with their arms folded across their chest and stood up five times consecutively as quickly as possible. The time taken to complete this maneuver was measured.

These tasks were assigned scores from 0 to 4 according to the established methods. The scores from the three tasks were added to obtain the total SPPB score (range: 0–12). A higher score indicated better physical performance.

### Statistical analysis

Continuous variables such as patient characteristics and clinical parameters were expressed as mean ± standard deviation. Categorical variables were expressed as numbers (percentages). Normality of distribution for continuous variables was evaluated using the Shapiro–Wilk test. Patients with HF were divided into two groups based on readmission within the past year. Baseline characteristics and physical function were compared using the Mann–Whitney U test and χ^2^ test. Logistic regression analysis was performed to evaluate whether patient characteristics, clinical parameters, and physical function at discharge were associated with readmission within the past year. A model was constructed using a stepwise forward selection procedure based on Akaike’s information criterion. The explanatory variables used in this model were those that showed statistical significance in the univariate analysis (P <0.05).

All statistical analyses were performed using EZR (Saitama Medical Center, Jichi Medical University, Saitama, Japan), a graphical user interface for R (R Core Team, 2022). Specifically, it is a modified version of R commander designed to add statistical functions frequently used in biostatistics^21)^.

### Ethical consideration

This study was approved by the Ethics Committee of the Medical Corporation Tanakakai Musashigaoka Hospital (no. H30-3) and complied with the principles of the Declaration of Helsinki.

## Results

### Patient characteristics and clinical parameters

**Fig. 1.**
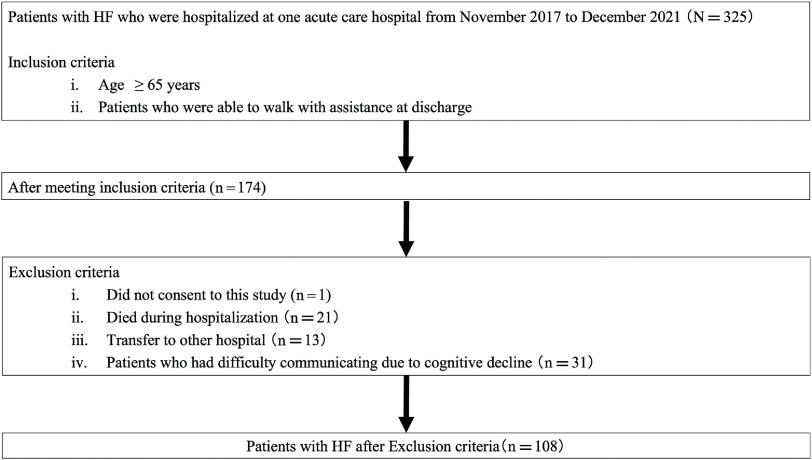
Flowchart of patient selection

A flowchart of patient selection is shown in Figure 1. Among 325 patients hospitalized with HF, 174 met the inclusion criteria. Sixty-six patients were excluded for not providing consent (n = 1), death during hospitalization (n = 21), transfer to another hospital (n = 13), and poor general condition that might have caused difficulties in measurements (n = 31). Finally, 108 patients were included and divided into the non-readmission group (n = 76) and readmission group (n = 32). Table 1 shows the clinical characteristics of all patients and the results of the comparison between the groups.

HF, heart failure

**Table 1.** Comparison between the two groups of clinical parameters, comorbidity, medication, cardiac function, laboratory data, nutrition, and physical function of all participants

	All (N = 108)	Non-readmission (n = 76)	Readmission (n = 32)	P-value
Clinical parameter				
Age (years)	85.5 ± 7.2	84.5 ± 7.8	87.7 ± 4.9	0.058
Sex (female), number (%)	54 (50.0)	41 (53.9)	19 (59.4)	0.292
BMI (kg/m^2^)	22.1 ± 4.2	22.1 ± 4.5	21.9 ± 3.5	0.719
Length of hospital stay (days)	30.0 ± 17.6	28.2 ± 16.5	34.4 ± 19.1	0.042
Initiation of rehabilitation (days)	6.0 ± 4.6	6.0 ± 5.1	5.9 ± 3.0	0.206
NYHA (I/II/III/IV), number	0/68/40/0	0/54/22/0	0/14/18/0	0.007
Comorbidity				
CCI	2.7 ± 1.3	2.5 ± 1.4	3.1 ± 1.0	0.008
HT, number (%)	91 (84.3)	61 (80.3)	30 (93.8)	0.090
DM, number (%)	71 (65.7)	49 (64.5)	22 (68.8)	0.825
Af, number (%)	66 (61.1)	42 (55.3)	24 (75.0)	0.082
Ischemic heart disease, number (%)	63 (58.3)	43 (56.6)	20 (62.5)	0.671
Valvular disease, number (%)	64 (59.3)	43 (56.6)	21 (65.6)	0.402
Medication				
β-blocker, number (%)	58 (53.7)	40 (52.6)	18 (52.3)	0.833
ACE-I/ARB, number (%)	54 (50.0)	41 (53.9)	13 (40.6)	0.292
Diuretic, number (%)	84 (77.8)	56 (73.7)	28 (87.5)	0.135
Cardiac function				
LVEF (%)	59.8 ± 14.1	59.6 ± 14.2	60.4 ± 13.8	0.724
Laboratory data				
BNP (pg/mL)	319.8 ± 225.8	284.0 ± 216.3	404.9 ± 225.0	0.010
Cr (g/dL)	1.3 ± 0.6	1.2 ± 0.5	1.4 ± 0.7	0.157
BUN (g/dL)	26.9 ± 13.3	26.1 ± 13.4	28.9 ± 13.0	0.142
eGFR (mL/min/1.73 m^2^)	42.7 ± 17.3	44.4 ± 17.5	38.7 ± 16.1	0.176
Hb (g/dL)	11.5 ± 1.9	11.6 ± 2.0	11.3 ± 1.7	0.514
Nutrition				
Alb (g/dL)	3.4 ± 0.5	3.4 ± 0.5	3.3 ± 0.4	0.060
GNRI	89.3 ± 8.9	89.9 ± 9.3	88.0 ± 7.8	0.180
Physical function				
Maximal QIS (Nm/kg)	0.8 ± 0.3	0.9 ± 0.3	0.7 ± 0.3	0.015
Grip strength (kg)	17.5 ± 6.1	18.3 ± 6.2	15.5 ± 5.5	0.034
SPPB	6.6 ± 3.6	7.5 ± 3.5	4.5 ± 3.0	<0.001
Values are presented as mean ± standard deviation or number (percentage).				
BMI, body mass index; NYHA, New York Heart Association functional classification; CCI, Charlson comorbidity index; HT, high blood pressure; DM, diabetes mellitus; Af, atrial fibrillation; ACE-I, angiotensin-converting enzyme inhibitor; ARB, angiotensin II receptor blocker; LVEF, left ventricular ejection fraction; BNP, brain natriuretic peptide; Cr, creatinine; BUN, blood urea nitrogen; eGFR, estimated glomerular filtration rate; Hb, hemoglobin; Alb, albumin; GNRI, Geriatric Nutritional Risk Index; QIS, quadriceps isometric strength; SPPB, Short Physical Performance Battery				

The mean age of the patients was 85.5 ± 7.2 years, and 50% were female. Altogether, 34 patients were over 90 years of age. There were no significant differences in age, sex, BMI, and time from admission to the start of rehabilitation between the readmission and non-readmission groups. Patients in the readmission group had a significantly longer hospital stay compared to those in the non-readmission group. All patients enrolled in this study were classified into NYHA classes II and III. The readmission group exhibited significantly higher severity of NYHA class than the non-readmission group.

Significant differences were observed in the CCI scores between the groups. However, the incidence of comorbidities such as hypertension, diabetes, atrial fibrillation, valvular disease, and ischemic heart disease was not significantly different between the groups.

No significant differences were observed in the use of beta-blockers, angiotensin-converting enzyme inhibitors, angiotensin II receptor blockers, or diuretics between the readmission and non-readmission groups.

No significant differences were observed in the LVEF, Cr level, BUN level, or eGFR. However, BNP was significantly higher in the readmission group than in the non-readmission group. No significant differences were observed in the Hb level, Alb level, or GNRI between the groups.

The maximal QIS, grip strength, and SPPB were significantly lower in the readmission group than in the non-readmission group.

### Independent factors associated with readmission

Table 2 shows the results of the logistic regression analysis. The NYHA class, BNP level, maximal QIS, grip strength, and SPPB score were selected as explanatory variables and analyzed using stepwise selection. BNP level and SPPB score were identified as independent factors associated with readmission within the past year.

**Table 2.** Odds ratio of factors related to hospital readmission by logistic regression analysis

	Univariate analysis			Multivariate analysis		
	OR	95% CI	P-value	OR	95% CI	P-value
Length of hospital stay	1.020	0.996–1.040	0.100			
NYHA	3.160	1.340–7.430	0.008			
CCI	1.330	0.977–1.820	0.069			
BNP	1.000	1.000–1.000	0.013	1.000	1.000–1.000	0.039
Maximal QIS	0.123	0.024–0.623	0.011			
Grip strength	0.923	0.857–0.995	0.036			
SPPB	0.773	0.674–0.886	<0.001	0.776	0.674–0.895	<0.001
OR, odds ratio; CI, confidence interval; NYHA, New York Heart Association; CCI, Charlson comorbidity index; BNP, brain natriuretic peptide; QIS, quadriceps isometric strength; SPPB, Short Physical Performance Battery						

## Discussion

In the present study, we examined the factors associated with readmission of patients with HF within the past year in Japan, which is becoming a super-aging society. Reportedly, ADLs are associated with readmission in Japan, and the presence or absence of sarcopenia affects the prognosis^22–25)^. The readmission group in the present study exhibited higher NYHA class, more comorbidities, higher BNP levels, and lower physical function and physical performance than the non-readmission group. Furthermore, BNP level and SPPB score were identified as independent risk factors for readmission in the multivariate analysis. The mean age of patients in the present study was 85.5 ± 7.2 years, which was higher than that reported in previous studies^22–24)^. In Japan, no definite view has been obtained about the effect of cardiac rehabilitation on older patients with HF, and the influence of physical performance of older patients with HF on the risk of readmission within the past year is unknown. The main finding of the present study was that physical performance affected the risk of readmission within the past year in older patients with HF having an average age of 85 years or above, suggesting the importance of cardiac rehabilitation in these patients.

BNP is a cardiac hormone synthesized in the ventricles and a biomarker that sensitively reflects the load on the ventricles^26–29)^. Previous studies have reported that a high BNP level at discharge is associated with higher readmission rates and mortality^30)^. BNP is secreted by the myocardium in response to wall stress, and it is affected by various factors. For example, obesity lowers the BNP levels, whereas aging, atrial fibrillation, and worsening renal function increase the BNP levels^31–34)^. There was no significant difference in BMI and renal function between the readmission non-readmission groups in the present study. In addition, BNP level was found to be an independent risk factor for readmission in the multivariate analysis. However, the odds ratio was 1.000 (95% confidence interval: 1.000–1.000), and further research is needed to determine how BNP affects readmission in patients with HF.

Patients with HF having low physical performance are more likely to be readmitted due to the increased cardiac load required for daily activities after discharge. Additionally, patients with HF may have decreased physical performance after discharge and may be readmitted due to events such as cardiovascular diseases, respiratory diseases, exacerbation of orthopedic diseases, and falls^22)^. Previous studies have reported a relationship between readmission and ADLs^22)^, which is influenced by physical performance (such as that determined using the SPPB score) and may result in a decline in ADLs due to inactivity caused by decreased physical capacity. A previous study reported that the SPPB score is a useful predictor of future decline in ADLs among older adults^17)^. Therefore, the SPPB score at discharge is considered an important factor for predicting the risk of readmission. Long-term inactivity during hospitalization makes daily life after discharge difficult due to deterioration of physical function including muscle strength and exercise tolerance and concomitant deterioration of ADLs. Therefore, it is important to improve physical function, physical performance, and ADLs by introducing cardiac rehabilitation during acute treatment of patients to reduce the risk of readmission.

In Japan, people aged ≥90 years are defined as “super-elderly”^35)^. The present study included 34 patients aged ≥90 years, accounting for more than 30% of all patients. Our data may be important for preventing readmission among older adults with HF. Reports of early readmission (90–180 days) after discharge have increased over the past few years^22,24,36)^, but very few studies have followed patients for longer periods. It is clear that Japanese patients with HF are at a high risk of readmission within 1 year^13)^. The readmission rates in previous studies were 5.4% within 30 days, approximately 20% within 90 days, approximately 24% within 180 days, and 26%–35% within 1 year; and the risk of readmission increased with increasing observation period^10,11,22,36)^. Therefore, continued follow-up after preventing early readmission is important. Moreover, results of the present study suggest the importance of cardiac rehabilitation to maintain physical performance. However, in Japan, there is insufficient evidence on the effectiveness of cardiac rehabilitation in older patients with HF, and very few studies have investigated the relationship between physical performance and readmission. Thus, further research is required on this topic.

### Limitations

This study has some limitations. It was a single-center study with a small sample size. To avoid sampling bias, we randomly selected participants from both the groups. Based on the inclusion and exclusion criteria, only 33.2% of the patients with HF who were hospitalized for acute exacerbations were included in this study. Therefore, the results of this study may not be generalizable to other patients with severe HF. A detailed evaluation of frailty and body composition could not be conducted. Frailty and sarcopenia have been shown to affect the prognosis of patients with HF^37,38)^. Therefore, detailed assessment of frailty and sarcopenia may be necessary. The present study did not evaluate left ventricular diastolic function. The effects of cardiac rehabilitation interventions have not been investigated. Determining the effects of cardiac rehabilitation is an important issue in Japan, where the average age of the population is expected to increase in the future. Studies that consider the interventional effect of cardiac rehabilitation are required to prevent readmission.

## Conclusion

Patients who were readmitted due to exacerbation of HF within the past year exhibited longer hospital stays, multiple comorbidities, higher NYHA class, higher BNP levels, and lower physical performance than those from the non-readmission group. In addition, the multivariate analysis showed that BNP level and SPPB score were associated with readmission for HF.

## Acknowledgments

The authors would like to thank Shu Tsuruzoe, Shingo Oda, Keiji Ushijima, Hiromichi Yuki, Shinichi Kihara, Ren Fujii, Takashi Kamakura, Mitsuru Matsumura, Soichiro Maeda, Yu Katsuragi, and Ayano Omura. The authors would also like to thank all the members of the rehabilitation team at Musashigaoka Hospital, all the members of the Oike Laboratory of the Graduate School of Kyushu University of Nursing and Welfare, and all the members of Tabira Laboratory, Graduate School of Kio University.

## Conflict of Interest

The authors report no conflicts of interest.

## References

[1] Cabinet Office Japan [Internet]. Annual Report on the Aging Society [Summary July] FY2020. 2020 Jul [cited 2022 Mar 9]; [About 3 p.]. Available from: https://www8.cao.go.jp/kourei/english/annualreport/2020/pdf/2020.pdf

[2] Santulli G, Ciccarelli M, *et al.*: Physical activity ameliorates cardiovascular health in elderly subjects: the functional role of the β adrenergic system. Front Physiol. 2013; 4: 209. 23964243 10.3389/fphys.2013.00209 PMC3740240

[3] Go AS, Mozaffarian D, *et al.*: Heart disease and stroke statistics—2014 update: a report from the American Heart Association. Circulation. 2014; 129: e28–e292. 24352519 10.1161/01.cir.0000441139.02102.80 PMC5408159

[4] Okura Y, Ramadan MM, *et al.*: Impending epidemic: future projection of heart failure in Japan to the year 2055. Circ J. 2008; 72: 489–491. 18296852 10.1253/circj.72.489

[5] Jencks SF, Williams MV, *et al.*: Rehospitalizations among patients in the Medicare fee-for-service program. N Engl J Med. 2009; 360: 1418–1428. 19339721 10.1056/NEJMsa0803563

[6] Owan TE, Hodge DO, *et al.*: Trends in prevalence and outcome of heart failure with preserved ejection fraction. N Engl J Med. 2006; 355: 251–259. 16855265 10.1056/NEJMoa052256

[7] Butrous H, Hummel SL: Heart failure in older adults. Can J Cardiol. 2016; 32: 1140–1147. 27476982 10.1016/j.cjca.2016.05.005 PMC5503696

[8] Dunlay SM, Redfield MM, *et al.*: Hospitalizations after heart failure diagnosis: a community perspective. J Am Coll Cardiol. 2009; 54: 1695–1702. 19850209 10.1016/j.jacc.2009.08.019 PMC2803107

[9] Deeka H, Skouri H, *et al.*: Readmission rates and related factors in heart failure patients: a study in Lebanon. Collegian. 2016; 23: 61–68. 27188041 10.1016/j.colegn.2014.11.001

[10] Shiraishi Y, Kohsaka S, *et al.*: 9-year trend in the management of acute heart failure in Japan: a report from the National Consortium of Acute Heart Failure Registries. J Am Heart Assoc. 2018; 7: e008687. 30371201 10.1161/JAHA.118.008687 PMC6222932

[11] Tsutsui H, Tsuchihashi-Makaya M, *et al.*: Clinical characteristics and outcomes of hospitalized patients with heart failure in Japan: rationale and design of Japanese Cardiac Registry of Heart Failure in Cardiology (JCARE-CARD). Circ J. 2006; 70: 1617–1623. 17127810 10.1253/circj.70.1617

[12] Kitagawa T, Oda N, *et al.*: Hospitalization and medical cost of patients with elevated serum N-terminal pro-brain natriuretic peptide levels. PLoS One. 2018; 13: e0190979. 29304158 10.1371/journal.pone.0190979 PMC5755927

[13] Tamaki Y, Kazawa K, *et al.*: Characteristics of heart failure patients incurring high medical costs via matching specific health examination results and medical claim data: a cross-sectional study. BMJ Open. 2019; 9: e031422. 10.1136/bmjopen-2019-031422 PMC6924797 31843826

[14] Guralnik JM, Ferrucci L, *et al.*: Lower-extremity function in persons over the age of 70 years as a predictor of subsequent disability. N Engl J Med. 1995; 332: 556–562. 7838189 10.1056/NEJM199503023320902 PMC9828188

[15] Pavasini R, Guralnik J, *et al.*: Short physical performance battery and all-cause mortality: systematic review and meta-analysis. BMC Med. 2016; 14: 215. 28003033 10.1186/s12916-016-0763-7 PMC5178082

[16] Yuguchi S, Saitoh M, *et al.*: Impact of preoperative frailty on regaining walking ability in patients after cardiac surgery: multicenter cohort study in Japan. Arch Gerontol Geriatr. 2019; 83: 204–210. 31082565 10.1016/j.archger.2019.04.003

[17] Wang DXM, Yao J, *et al.*: Muscle mass, strength, and physical performance predicting activities of daily living: a meta-analysis. J Cachexia Sarcopenia Muscle. 2020; 11: 3–25. 31788969 10.1002/jcsm.12502 PMC7015244

[18] Tsutsui H, Isobe M, *et al.*: JCS2017/JHFS guideline on diagnosis and treatment of acute and chronic heart failure: digest version. Circ J. 2019; 83: 2084–2184. 31511439 10.1253/circj.CJ-19-0342

[19] JCS/JHFS [Internet]. Guideline Focused Update on Diagnosis and Treatment of Acute and Chronic Heart Failure, 2021 [updated 2021 Sep 10; cited 2022 Mar 9]; [About 11 p.]. Available from: https://www.j-circ.or.jp/cms/wp-content/uploads/2021/03/JCS2021_Tsutsui.pdf (in Japanese)

[20] Japan Sports Agency [Internet]. Shintairyoku test jisshiyoko (65–79 sai taisho) [cited 2022 June 10]; [About 3 p.]. Available from: https://www.mext.go.jp/sports/content/20220329-spt_kensport01-300000992_05.pdf (in Japanese)

[21] Kanda Y: Investigation of the freely available easy-to-use software ‘EZR’ for medical statistics. Bone Marrow Transplant. 2013; 48: 452–458. 23208313 10.1038/bmt.2012.244 PMC3590441

[22] Kitamura M, Izawa KP, *et al.*: Relationship between activities of daily living and readmission within 90 days in hospitalized elderly patients with heart failure. BioMed Res Int. 2017; 2017: 7420738. 29201912 10.1155/2017/7420738 PMC5671682

[23] Takabayashi K, Kitaguchi S, *et al.*: A decline in activities of daily living due to acute heart failure is an independent risk factor of hospitalization for heart failure and mortality. J Cardiol. 2019; 73: 522–529. 30598389 10.1016/j.jjcc.2018.12.014

[24] Kitamura M, Izawa KP, *et al.*: Relationship among activities of daily living, nutritional status, and 90 day readmission in elderly patients with heart failure. Int J Environ Res Public Health. 2019; 16: 5068. 31842307 10.3390/ijerph16245068 PMC6950285

[25] Konishi M, Kagiyama N, *et al.*: Impact of sarcopenia on prognosis in patients with heart failure with reduced and preserved ejection fraction. Eur J Prev Cardiol. 2021; 28: 1022–1029. 33624112 10.1093/eurjpc/zwaa117

[26] Mukoyama M, Nakao K, *et al.*: Brain natriuretic peptide as a novel cardiac hormone in humans: evidence for an exquisite dual natriuretic peptide system, atrial natriuretic peptide and brain natriuretic peptide. J Clin Invest. 1991; 87: 1402–1412. 1849149 10.1172/JCI115146 PMC295184

[27] Yoshimura M, Yasue H, *et al.*: Different secretion patterns of atrial natriuretic peptide and brain natriuretic peptide in patients with congestive heart failure. Circulation. 1993; 87: 464–469. 8425293 10.1161/01.cir.87.2.464

[28] Yasue H, Yoshimura M, *et al.*: Localization and mechanism of secretion of B-type natriuretic peptide in comparison with those of A-type natriuretic peptide in normal subjects and patients with heart failure. Circulation. 1994; 90: 195–203. 8025996 10.1161/01.cir.90.1.195

[29] Maisel AS, Nakao K, *et al.*: Japanese-Western consensus meeting on biomarkers. Int Heart J. 2011; 52: 253–265. 22008432 10.1536/ihj.52.253

[30] Fukuda H, Suwa H, *et al.*: Non-linear equation using plasma brain natriuretic peptide levels to predict cardiovascular outcomes in patients with heart failure. Sci Rep. 2016; 6: 37073. 27845390 10.1038/srep37073 PMC5109227

[31] Knudsen CW, Omland T, *et al.*: Impact of atrial fibrillation on the diagnostic performance of B-type natriuretic peptide concentration in dyspneic patients: an analysis from the breathing not properly multinational study. J Am Coll Cardiol. 2005; 46: 838–844. 16139134 10.1016/j.jacc.2005.05.057

[32] Redfield MM, Rodeheffer RJ, *et al.*: Plasma brain natriuretic peptide concentration: impact of age and gender. J Am Coll Cardiol. 2002; 40: 976–982. 12225726 10.1016/s0735-1097(02)02059-4

[33] Forfia PR, Watkins SP, *et al.*: Relationship between B-type natriuretic peptides and pulmonary capillary wedge pressure in the intensive care unit. J Am Coll Cardiol. 2005; 45: 1667–1671. 15893185 10.1016/j.jacc.2005.01.046

[34] McCullough PA, Duc P, *et al.*: B-type natriuretic peptide and renal function in the diagnosis of heart failure: an analysis from the breathing not properly multinational study. Am J Kidney Dis. 2003; 41: 571–579. 12612980 10.1053/ajkd.2003.50118

[35] Ouchi Y, Rakugi H, *et al.*: Redefining the elderly as aged 75 years and older: proposal from the Joint Committee of Japan Gerontological Society and the Japan Geriatrics Society. Geriatr Gerontol Int. 2017; 17: 1045–1047. 28670849 10.1111/ggi.13118

[36] Iwata K, Kitai T, *et al.*: Clinical impact of functional independent measure (FIM) on 180-day readmission and mortality in elderly patients hospitalized with acute decompensated heart failure. Heart Vessels. 2021; 36: 1536–1541. 33834270 10.1007/s00380-021-01841-y

[37] Kanenawa K, Isotani A, *et al.*: The impact of frailty according to clinical frailty scale on clinical outcome in patients with heart failure. ESC Heart Fail. 2021; 8: 1552–1561. 33547759 10.1002/ehf2.13254 PMC8006666

[38] Curcio F, Testa G, *et al.*: Sarcopenia and heart failure. Nutrients. 2020; 12: 211. 31947528 10.3390/nu12010211 PMC7019352

